# Polarization-resolved microscopy reveals a muscle myosin motor-independent mechanism of molecular actin ordering during sarcomere maturation

**DOI:** 10.1371/journal.pbio.2004718

**Published:** 2018-04-27

**Authors:** Olivier Loison, Manuela Weitkunat, Aynur Kaya-Çopur, Camila Nascimento Alves, Till Matzat, Maria L. Spletter, Stefan Luschnig, Sophie Brasselet, Pierre-François Lenne, Frank Schnorrer

**Affiliations:** 1 Aix Marseille Université, CNRS, IBDM, Marseille, France; 2 Max Planck Institute of Biochemistry, Muscle Dynamics Group, Martinsried, Germany; 3 Institute of Neurobiology and Cells-in-Motion Cluster of Excellence (EXC 1003 – CiM), University of Münster, Münster, Germany; 4 Department of Zoology, University of British Columbia, Vancouver, Canada; 5 Aix Marseille Université, CNRS, Centrale Marseille, Institut Fresnel, Marseille, France; University of Oxford, United Kingdom of Great Britain and Northern Ireland

## Abstract

Sarcomeres are stereotyped force-producing mini-machines of striated muscles. Each sarcomere contains a pseudocrystalline order of bipolar actin and myosin filaments, which are linked by titin filaments. During muscle development, these three filament types need to assemble into long periodic chains of sarcomeres called myofibrils. Initially, myofibrils contain immature sarcomeres, which gradually mature into their pseudocrystalline order. Despite the general importance, our understanding of myofibril assembly and sarcomere maturation in vivo is limited, in large part because determining the molecular order of protein components during muscle development remains challenging. Here, we applied polarization-resolved microscopy to determine the molecular order of actin during myofibrillogenesis in vivo. This method revealed that, concomitantly with mechanical tension buildup in the myotube, molecular actin order increases, preceding the formation of immature sarcomeres. Mechanistically, both muscle and nonmuscle myosin contribute to this actin order gain during early stages of myofibril assembly. Actin order continues to increase while myofibrils and sarcomeres mature. Muscle myosin motor activity is required for the regular and coordinated assembly of long myofibrils but not for the high actin order buildup during sarcomere maturation. This suggests that, in muscle, other actin-binding proteins are sufficient to locally bundle or cross-link actin into highly regular arrays.

## Author summary

Muscles produce high mechanical forces to power our daily movements. The mini-machines in our muscles that produce these forces are called sarcomeres. Every sarcomere consists of three protein filaments: thin actin filaments and thick myosin filaments that are linked by titin filaments. These three filaments are present in extremely high order in mature sarcomeres, resembling a liquid crystal. How such a crystalline-like molecular order is generated during sarcomere development is not well understood. Here, we applied a new microscopy technique—polarization-resolved microscopy, which was developed to quantify molecular order of molecules—to the flight muscles of the fruit fly *Drosophila melanogaster*, which is amenable to genetic manipulations. Using this new technique, we demonstrate that actin is indeed assembled into high molecular order during sarcomere development. We tested different mechanisms and found a role for myosin motors during the early assembly steps. However, the high-order organization of actin still happens in the absence of muscle myosin, suggesting that additional proteins other than myosin are involved in ordering actin during sarcomere development.

## Introduction

Most animals use muscles to produce the forces that power active body movements. Although the various striated muscle types—including vertebrate heart and skeletal muscles, as well as the numerous insect body muscles—differ significantly in their development and physiology [1,2], they all share the same basic organizational principle of their contractile mini-machines called sarcomeres. Each sarcomere is bordered by two Z-discs, anchoring the cross-linked plus (barbed) ends of highly regular actin filaments. The minus (pointed) ends of these actin filaments point towards the center of the sarcomere, where they can interact with motor domains of centrally located bipolar myosin filaments. Actin and myosin filaments are stably connected by a third, connecting filament composed of the gigantic protein titin. Titin’s N-terminus is anchored at the Z-disc, whereas its C-terminus connects to the myosin filaments in insect sarcomeres or extends all the way to the M-line in the middle of the myosin filaments in vertebrate sarcomeres [3–6]. Not only the structural organization of sarcomeres but also their molecular components are highly conserved from worms to humans, suggesting that the sarcomere represents an ancient molecular machine [7,8].

Sarcomeres are stereotyped units with a defined length, ranging from 3.0 to 3.4 μm in different relaxed human muscle fibers in vivo [9,10]. Muscle fibers of large animals need to be several centimeters long to effectively move the relative position of two neighboring bones. Therefore, hundreds of sarcomeric units need to assemble into long periodic chains, called myofibrils, which span the entire muscle fiber and thus mechanically link two skeletal elements. How muscle fibers solve this “dimension problem” during myofibrillogenesis is not well understood [3].

*Drosophila* adult indirect flight muscles have a mature muscle fiber length of about 1 mm and a resting sarcomere length of 3.2 μm in vivo. Using this developmental model system, three interesting observations have been made previously. First, laser-induced microlesion experiments revealed a buildup of mechanical tension in the developing myotube. This tension buildup precedes the formation of immature myofibrils and is essential for their regular formation [11]. Second, live imaging of endogenous muscle myosin heavy chain showed a simultaneous assembly of immature myofibrils throughout the entire muscle fiber. These myofibrils display an initial periodicity of about 1.8 μm [11]. Third, during the next 3 days of development, the sarcomere periodicity increases to 3.2 μm present in adult flight muscles [11–13]. Together, these observations suggested that mechanical tension drives the self-organization of actin, myosin, and titin filaments into immature myofibrils [3,11]. However, how this self-organization is achieved molecularly and how immature myofibrils mature to their final pseudocrystalline order are poorly understood.

One prominent model, the premyofibril model, proposes a stepwise mode of myofibrillogenesis. In a first step, nonmuscle myosin II is proposed to assemble early “premyofibrils.” In a second step, nonmuscle myosin is replaced by muscle myosin to enable myofibril maturation [14–16]. However, whether and how the myosin isoforms actively contribute to the formation of actin order during myofibrillogenesis is not clear. This gap in knowledge is mainly due to two reasons. First, functional in vivo data are sparse because genetic loss-of-function studies of nonmuscle myosin in mice are complicated by redundancies [17,18]. Second, light or electron microscopic images cannot easily resolve the molecular order of proteins, which is high in mature sarcomeres but much lower in early myotubes. Thus, the molecular order analysis of sarcomeric proteins in genetic mutants in vivo is challenging.

In this study, we investigate the mechanism of myofibrillogenesis by combining functional genetics of myosin isoforms with polarization-resolved microscopy, a newly developed microscopy technique that allows us to detect molecular order. Polarization-resolved microscopy was developed to reveal local order of lipids in cell membranes in vitro [19] and proteins such as actin at the cell cortex of early *Drosophila* embryos [20]. We apply it here to investigate actin order generation during muscle development in vivo. We measure molecular actin order during various steps of myofibrillogenesis and test for a genetic requirement of both nonmuscle and muscle myosin during actin ordering. We find a gain in molecular actin order before a reliable periodic actin pattern is detectable by light microscopy. This order gain indeed depends on both nonmuscle and muscle myosin function. We identify a steep increase in molecular actin order during sarcomere maturation, which is largely independent of muscle myosin or its motor activity. These results suggest an important role for actin cross-linkers other than myosin in local actin ordering during sarcomere and myofibril maturation.

## Results

### Determination of molecular actin order by polarization-resolved microscopy

**Fig 1:**
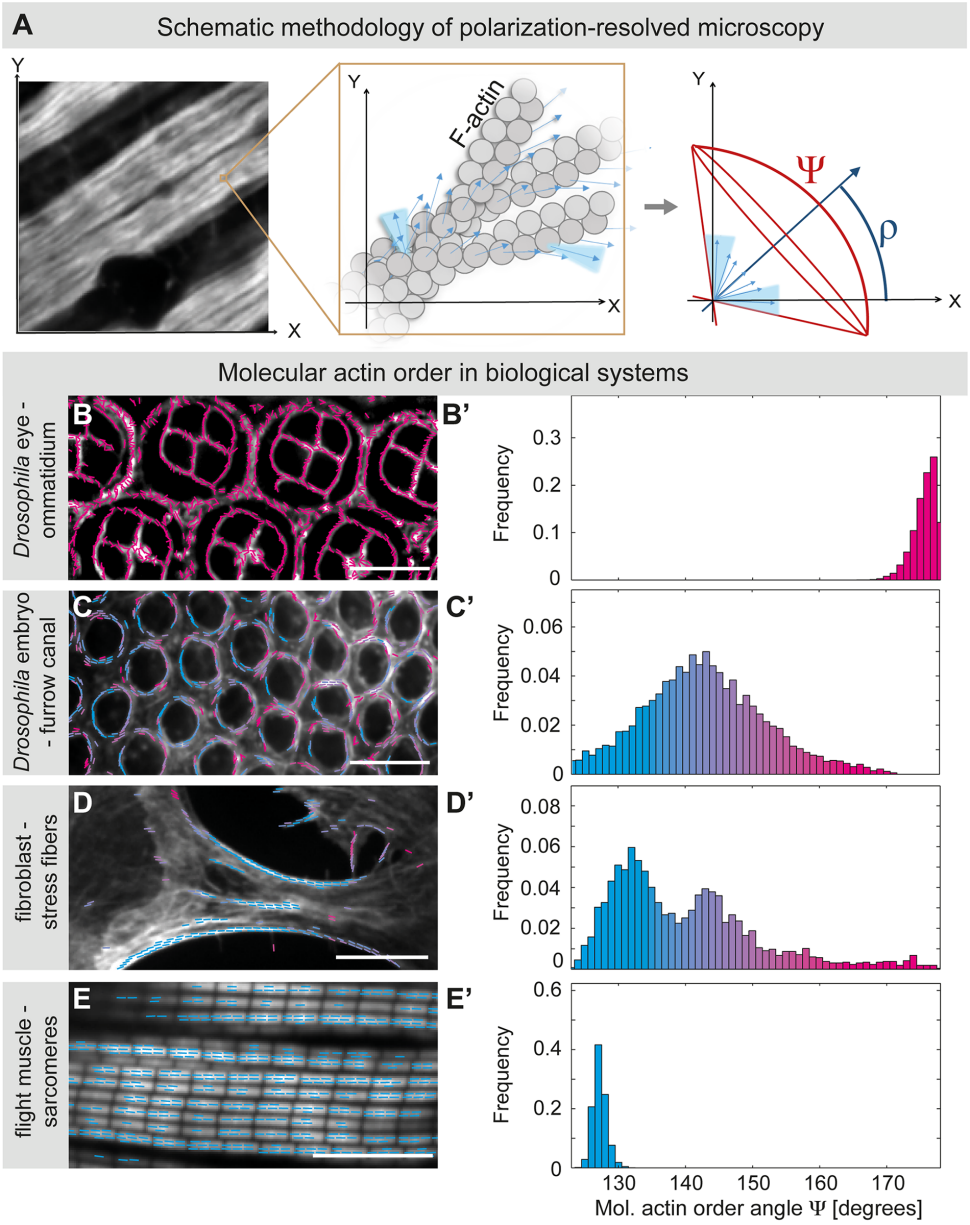
Polarization-resolved microscopy—A method to determine actin order in biological systems. (A) Polarization-resolved microscopy provides information about F-actin molecular order Ψ and the average actin filament orientation ρ. The left image shows a 32-h APF flight muscle stained with phalloidin–Alexa488 and overlaid with an orange box representing the confocal volume. The middle image displays a hypothetical actin order in the confocal volume, with the different orientations of the phalloidin–Alexa488 dipoles shown by blue arrows. For each confocal volume, one value for the actin molecular order Ψ can be determined. The right diagram shows the average actin filament orientation angle ρ in blue and the actin molecular order angle Ψ in red for the hypothetical confocal volume shown. All order angle values Ψ are shifted by a term Ψ_0_, as first, the Alexa488 dipole forms an undefined angle to the helicoidal actin filament (blue arrows) and second, the Alexa488 label is connected to phalloidin with a linker, allowing some molecular flexibility (represented by the solid light blue cones in the Fig). An order angle Ψ = Ψ_0_ is thus a signature of perfect order, whereas a random distribution corresponds to Ψ = 180°. (B–E) Molecular actin order in different biological systems. Samples were fixed and stained with the same phalloidin–Alexa488 probe to reveal a highly disordered actin cytoskeleton in the developing *Drosophila* pupal eye (41-h APF; several ommatidia are shown) (panels B, B’; Ψ close to 180°), a more regular actin cytoskeleton in the furrow canal of early *Drosophila* embryos (panels C, C’; Ψ = 140°–150°), and in stress fibers of plated mouse NIH-3T3 fibroblasts (panels D, D’; Ψ = 130°–150°), compared with a very-high actin order in mature sarcomeres of 90-h APF flight muscles (panels E, E’; Ψ = 127°). The colors in the overlaid images (panels B–E) relate with the colors in the order tables (B’–E’); the bluer, the higher the molecular actin order. Scale bars represent 10 μm. See S1 Data for primary data. APF, After Puparium Formation; F-actin, filamentous actin.

To get insight into the molecular organization of actin during myofibril and sarcomere formation in *Drosophila* flight muscles, we applied polarization-resolved microscopy. During polarization-resolved microscopy, a series of confocal images is acquired with variable angles of polarized excitation light. The maximum fluorescence is achieved when the polarization angle of the excitation light matches the dipole angles of the fluorophores [19] (see Materials and methods for details). Therefore, if all fluorophores have the same dipole orientation and thus a high molecular order, a large modulation of the fluorescent signal is detected when the polarization axis of the excitation light is rotated. From this modulation, polarization-resolved microscopy quantifies two parameters of molecular order for the measured fluorophores: their mean orientation (ρ) and their angular distribution width (Ψ) around this mean orientation. Thus, Ψ is a proxy for molecular order of the measured fluorophores. Both ρ and Ψ values are determined for each pixel (Fig 1A). Entirely disordered fluorophores result in a Ψ of 180°; however, even a crystalline-ordered protein does not result in a Ψ of close to 0° because fluorophores generally exhibit a remanent orientational disorder due to the flexibility of the linker to the protein of interest.

In order to apply this method to quantify actin order, we opted for phalloidin coupled with Alexa488, which has been shown to exhibit the least flexibility compared with other labels [21]. Phalloidin binds selectively to filamentous actin (F-actin), and therefore the order of the different actin filaments in the confocal volume can be compared within one image and between different samples (Fig 1A).

For proof of principle experiments of our set-up, we aimed to compare the order of F-actin in different biological systems. We find that F-actin in the ommatidia of the pupal *Drosophila* eyes is poorly ordered (Ψ = 170° to 180°; Fig 1B and 1B’), whereas the actin cables of the ingressing furrow canal during cellularization in the *Drosophila* embryo show significantly higher molecular order (Ψ = 140° to 150°; Fig 1C and 1C’), consistent with previous reports [20]. Actin order within stress fibers of plated adhesive fibroblasts can be even higher (Ψ = 130° to 150°; Fig 1D and 1D’) [20]. We found that actin filaments within the sarcomeres of mature flight muscles of 90-h After Puparium Formation (APF) pupae contain the highest detectable order of all systems tested here, with a very marked and narrow order peak (around Ψ = 127°; Fig 1E, S1 Data). Note that, under the signal-to-noise conditions obtained in the measurements, a precision of about 1° for Ψ is expected [19]. Considering the pseudocrystalline actin arrangement in mature flight muscles [22,23], the measured Ψ value of 127° should correspond to quasi-perfect order—its deviation from 0° likely results from the angle formed by the phalloidin probe on the actin helical backbone and the flexibility of the Alexa488 link to the probe (Fig 1A) [21]. Taken together, these data indicate that our labeling and imaging methods of actin filaments can indeed be used to quantify molecular actin order in various biological samples.

### Molecular actin order buildup during myofibrillogenesis and sarcomere maturation

**Fig 2:**
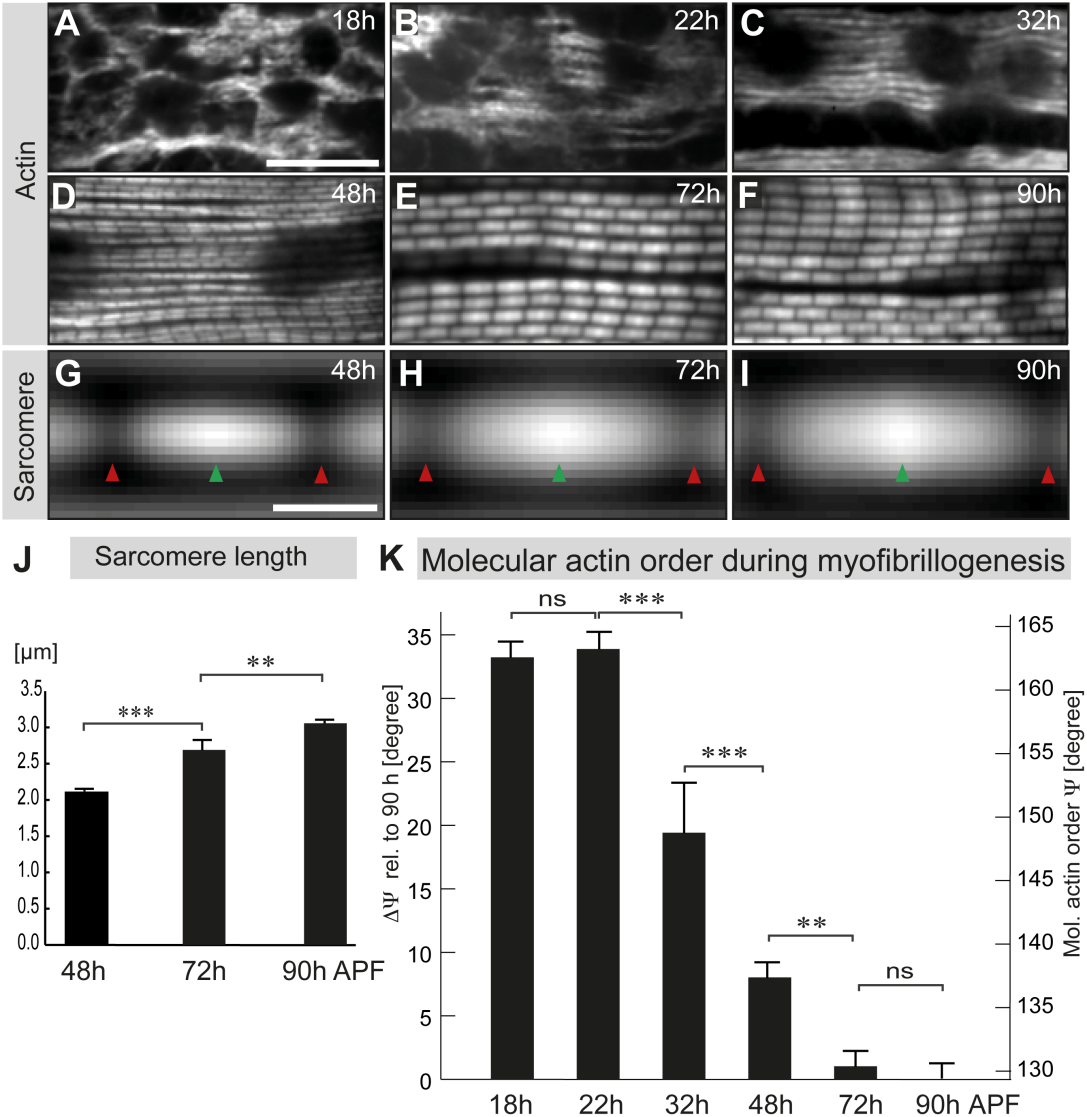
Polarization-resolved microscopy reveals the buildup of molecular actin order during sarcomerogenesis. (A–F) Flight muscle myofibrils at different developmental stages from 18-h to 90-h APF stained by Alexa488-labeled phalloidin and imaged with a spinning disc microscope. Scale bar represents 10 μm. (G–I) Averaged sarcomere actin concentration maps obtained after automated sarcomere detection. Arrowheads indicate M-line (red) and Z-disc (green) positions. Scale bar represents 1 μm. (J) Quantification of sarcomere length. *p*-values: **P < 0.01; ***P < 0.001 (nonparametric Mann-Whitney U test; see S1 Data). Error bars represent SD. (K) Molecular actin order angles determined in fixed developing flight muscles at different pupal stages. The same samples as in Fig 2 were used. The molecular disorder ΔΨ relative to highest order value at 90-h APF is shown. Note the strong gain of order from 22-h to 32-h APF and from 32-h to 48-h APF. Error bars represent SD. *p*-values: **P < 0.01; ***P < 0.001 (nonparametric Mann-Whitney U test; see S1 Data for primary data). APF, After Puparium Formation; ns, nonsignificant.

We next applied our method to determine F-actin order during different stages of flight muscle development. Imaging actin with the spinning disc channel confirmed that, at 18-h APF, myotubes have initiated attachment to tendons, and actin filaments form a loose meshwork, which progressively bundles into individual immature myofibrils at 32-h APF (Fig 2A–2C). During these stages, mechanical tension strongly increases in the myotube [11], but the immature myofibrils do not yet exhibit a clear periodic actin pattern at 32-h APF (Fig 2C). In contrast, from 48-h APF onwards, a periodic actin pattern is readily detected (Fig 2D–2F). Taking advantage of this periodicity, we averaged the intensity of multiple sarcomeres to determine the average position of M-line and Z-discs and to quantify sarcomeric growth from 48-h APF on (Fig 2G–2J). We found a sarcomere length of 2.1 μm at 48-h APF, which grows to 2.7 and 3.1 μm at 72-h and 90-h APF, respectively. These length measures are in agreement with previous reports [24] [13], validating our imaging and automated sarcomere detection pipeline. We found that the variability of sarcomere length within individual pupae is below 3% (SD), demonstrating a synchronous growth of all sarcomeres in all muscle fibers of each pupa. This strongly supports a self-organization mechanism that coordinates sarcomere maturation across the entire muscle fiber.

**Fig 3:**
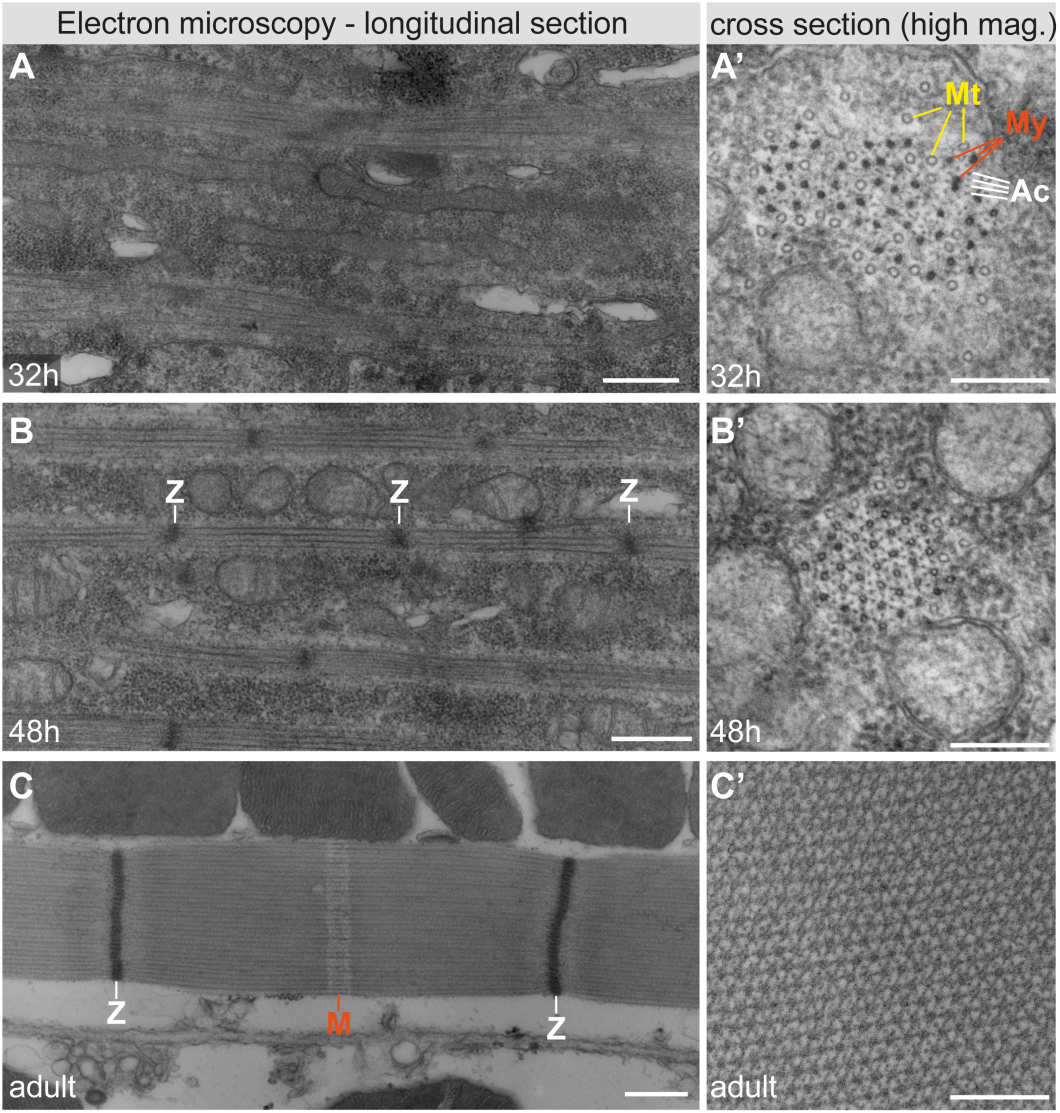
Electron microscopy of developing and adult flight muscle myofibrils. (A–C) Transmission electron microscopy images of longitudinal sections (panels A–C) and cross-sections (panels A’–C’) at 32-h APF (panels A, A’), 48-h APF (panels B, B’), and of adult flight muscles (panels C, C’). Note that myofibril periodicity is difficult to discern at 32-h APF; however, myosin filaments (dark circles; marked by “My” in orange) are next to actin filaments (small, lighter circles; “Ac” in white). Large, open circles are microtubules (“Mt” in yellow; panel A’). At 48-h APF, primitive Z-discs (“Z” in white; panel B) and regular actomyosin arrays are visible in the cross-section (panel B’). In 1-day-old adults, the Z-discs (white) and the M-line (red) are labeled (panel C). Scale bars represent 0.5 μm (panels A–C) and 0.2 μm (panels A’–C’). APF, After Puparium Formation.

In order to compare the molecular actin order between the different developmental stages, we used the angular distribution width of 90-h APF flight muscles (Ψ_90h_ = 129°; S1 Data) as a reference and determined the disorder of actin ΔΨ during development, as the deviation from this reference (ΔΨ = Ψ − Ψ_90h_). As expected, we find a large deviation of ΔΨ = 34° from the perfect order in the loose actin filament meshwork of 18-h and 22-h APF myotubes, which does not significantly decrease during that time (Fig 2K). However, from 22-h to 32-h APF, a period during which mechanical tension strongly increases and a first periodic muscle myosin pattern is detectable on the immature myofibrils [11], we observe a strong gain in molecular actin order, with ΔΨ decreasing by 14° (Fig 2K). These immature myofibrils do not yet show an obvious periodic actin pattern at 32-h APF (Fig 2C and [11]). However, the observed gain in molecular order strongly suggests that actin filaments begin to organize into the parallel polar organization present in actin filaments of mature sarcomeres. This is also supported by electron microscopic analysis of 32-h APF myotubes showing parallel actomyosin filaments (Fig 3A), with a regular spacing between actin and myosin filaments as revealed by cross-sections (Fig 3A’).

The actin order continues to increase dramatically, with ΔΨ reaching 8° at 48-h APF (Fig 2K). At this stage, the myofibrils display a regular periodic actin pattern, with a sarcomere length of 2.1 μm (Fig 2D and 2J), suggesting that actin filaments are already highly polarized and likely cross-linked at the developing Z-discs. This is further substantiated by electron microscopy, showing highly organized parallel actomyosin filaments at regular distances, interspaced with dense Z-discs at 48-h APF (Fig 3B and 3B’).

The actin order gain slows down until 72-h APF (ΔΨ decreasing by 7°), and no further statistically significant gain is detected until 90-h APF (Fig 2K, S1 Data), although the sarcomeres grow both in length and diameter (Figs 2J, 3C and 3C’). Together, these data demonstrate that molecular actin order in flight muscles increases during the formation of immature myofibrils at 32-h APF and is largely built up until 48-h APF, while during sarcomere elongation new actin subunits are added to already ordered actin filaments.

### Simultaneous buildup of molecular actin order during early stages of myofibrillogenesis

**Fig 4:**
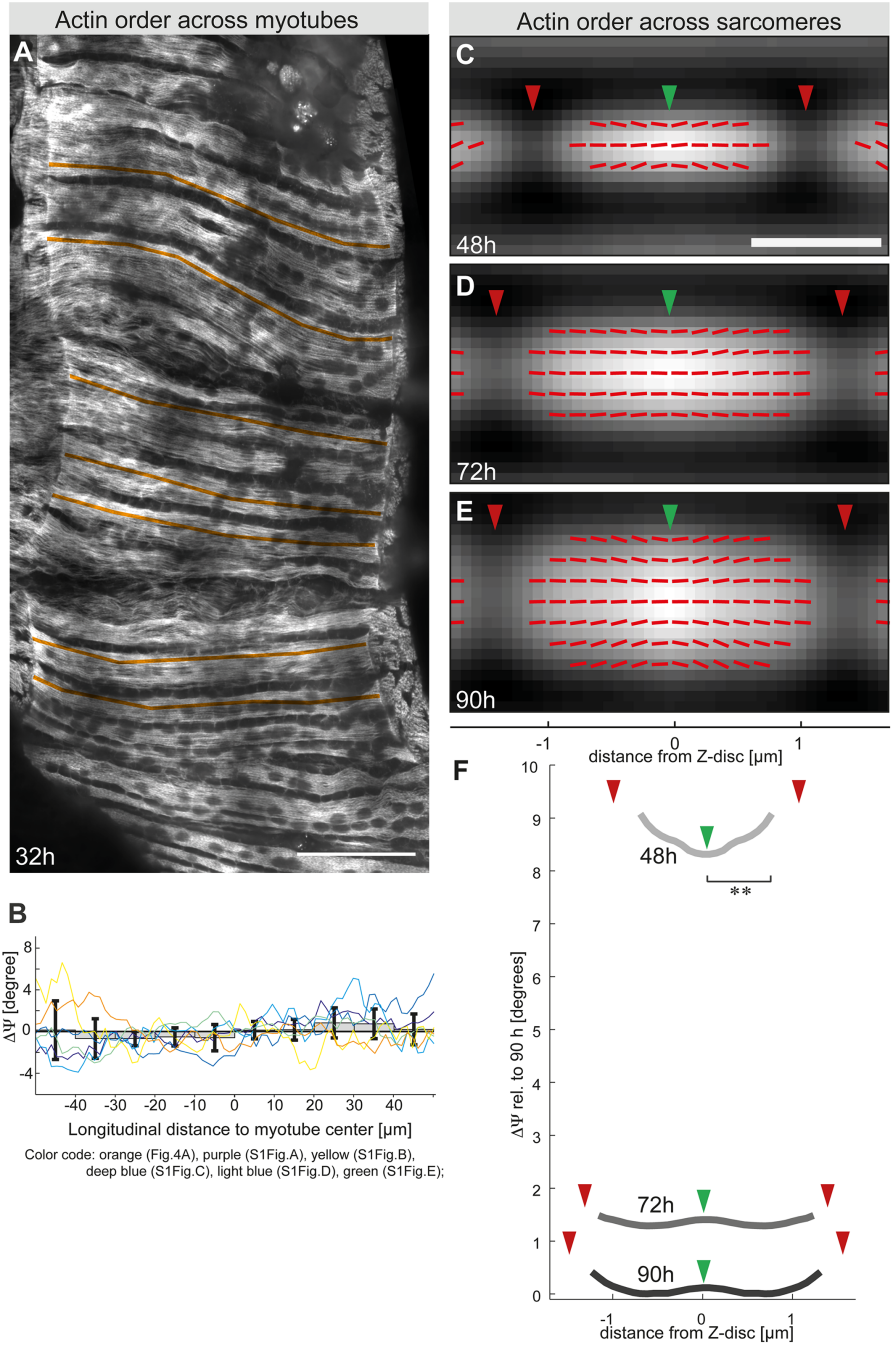
Simultaneous order buildup during myofibril initiation and subsarcomeric order during sarcomere maturation. (A) Flight muscle at 32-h APF stained with rhodamine-Alexa488. Overlaid longitudinal orange lines (from one fiber end to the other) indicate positions at which molecular actin order was determined. Scale bar represents 50 μm. (B) Molecular actin order angle longitudinal profile for flight muscles at 32-h APF. Each color line corresponds to an average profile obtained on an individual pupa (the orange one corresponds to the pupa shown in panel A; the others are displayed in S1 Fig). Bars indicate the mean values of 6 different pupae and error bars the SDs. Note that there is no significant difference between the center and the ends of the muscle fiber. Values are relative to the averaged value per pupa. (C–E) Grey maps showing the actin intensity of an averaged sarcomere with the Z-disc (green arrowhead) shown in the middle and the M-lines (red arrowhead) at 48-h (panel C), 72-h (panel D), and 90-h APF (panel E) as shown in Fig 2G–2I. Red sticks represent the local actin orientation angle ρ. Stick angles have been amplified 50 times relative to horizontal axis to visualize the small differences. Scale bar represents 1 μm. (F) Molecular actin order angle profiles along the averaged sarcomere length at 48-h (light grey), 72-h (dark grey), and 90-h APF (black). Values are relative to the minimum of the 90-h APF profile. Red arrows show M-line positions and green ones the Z-disc position. Note the relatively higher actin order at the developing Z-disc of 48-h APF sarcomeres. **P < 0.01; (Wilcoxon signed-rank test; see S1 Data for primary data). APF, After Puparium Formation.

Our earlier work suggested that immature myofibrils are built simultaneously throughout the developing flight muscle fibers [11]. However, observations from cultured cardiomyocytes in vitro rather indicated that early and mature myofibrils are present at the same time in the same cell [14,25]. To gain more in vivo data about this important step of myofibril initiation, we measured molecular actin order in flight muscles at 32-h APF, when the earliest immature myofibrils are detectable, at various positions within the large flight muscle fibers (Fig 4A, S1 Fig). We performed longitudinal line scans of actin order from one muscle fiber end to the other at various positions within the muscle fibers of 1 pupa and found that the molecular actin order varies by less than 4° when comparing the center of the myotube to its ends (Fig 4A and 4B). These data strongly suggest that molecular actin order and thus immature myofibrils are built up simultaneously throughout the entire muscle fiber.

### Actin order increases at the sarcomeric Z-disc during intermediate stages of myofibrillogenesis

From 48-h APF onwards, we could use our image analysis protocol to automatically detect individual sarcomeres of the large flight muscle fibers and compute averaged images of sarcomeres (see Fig 2G–2I). We took advantage of this protocol to investigate the relative molecular actin order within sarcomeres during sarcomere maturation. We find a significantly higher actin order at the Z-discs of developing sarcomeres at 48-h APF (Fig 4C and 4F). This may indicate that the actin filaments at the developing Z-discs are getting ordered first, supposedly by cross-linking to the forming Z-disc.

During sarcomere maturation after 48-h APF, the global actin order increases throughout the sarcomere until 90-h APF (Fig 4C–4F). Additionally, measuring the average actin orientation angle ρ at the micrometer scale shows that local actin orientation is homogeneously aligned along the sarcomere main axis, with only a slight deviation towards the inner region of the Z-discs (Fig 4C–4F). This is consistent with a slightly oblique orientation of thin filaments close to the Z-discs of adult flight muscles as seen by electron microscopy (Fig 3C). In conclusion, these data demonstrate that, despite slight differences in the speed of actin ordering within developing sarcomeres, high actin order is gained along the entire sarcomere during sarcomere maturation.

### Essential role for nonmuscle myosin during myofibrillogenesis

As the premyofibril model suggested a role for nonmuscle myosin II during early steps of myofibrillogenesis [14–16], we aimed to functionally test this hypothesis in vivo. Mutations in the single *Drosophila* nonmuscle myosin II heavy chain, *zipper* (*zip*), and in the nonmuscle myosin II regulatory light chain, *spaghetti-squash* (*sqh*), are embryonic lethal. A systematic RNA interference (RNAi) screen found that muscle-specific knock-down of *zip* or *sqh* results in late pupal lethality, suggesting a function of nonmuscle myosin in muscle [26]. We have reinvestigated the role of *zip* and *sqh* in muscle in more detail using additional RNAi hairpins and confirmed that 2 independent *sqh* RNAi and 3 independent *zip* RNAi lines result in late pupal lethality, with a few escaper flies being entirely flightless, when the hairpins are expressed during muscle development (S1 Table). This shows that nonmuscle myosin II is indeed required for normal muscle formation or function.

**Fig 5:**
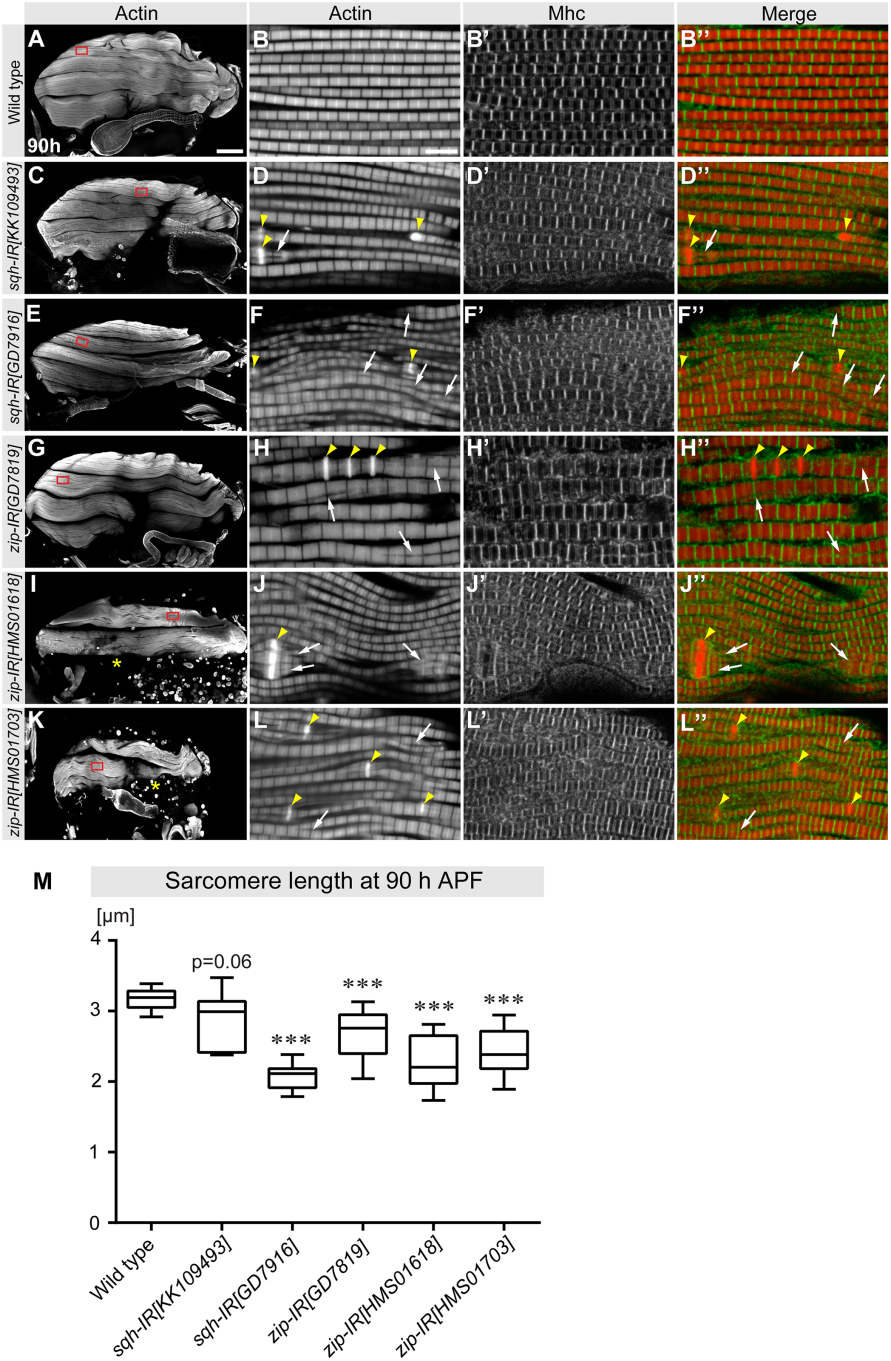
Nonmuscle myosin II is essential for ordered myofibrillogenesis at 90-h APF. (A–L) Hemithoraces of wild-type 90-h APF pupae and of the indicated *sqh* and *zip* knock-down genotypes (hairpins expressed with *Mef2*-GAL4) were stained with phalloidin (green) and anti-Mhc (red) and observed by confocal microscopy (panels A, C, E, G, I, and K). Missing flight muscle fibers after knock-down of *zip* are indicated with a yellow asterisk (panels I, K). Red boxes indicate the positions of the high-magnification images of the myofibrils. Actin accumulations are marked with yellow arrowheads and split myofibrils with white arrows. Scale bars represent 100 μm (panels A, C, E, G, I, and K) and 5 μm (panels B, D, F, H, J, and L). (M) Quantification of sarcomere length at 90-h APF of the indicated genotypes. Plots represent the average sarcomere length per pupa shown within the whiskers (minimum to maximum), with 50% of the values shown within the boxplots (25% to 75%) and the median indicated as a line. *p*-values: ***P < 0.001 (nonparametric Mann-Whitney U test; see S1 Data for primary data). APF, After Puparium Formation; Mhc, Myosin heavy chain; *sqh*, *spaghetti-squash*; *zip*, *zipper*.

To specifically test for a role of nonmuscle myosin during myofibrillogenesis, we examined flight muscle fiber, myofibril, and sarcomere morphology at the end of pupal development at 90-h APF. We find that upon *sqh* knock-down the fiber morphology is largely normal, whereas upon knock-down of *zip* some muscle fibers are missing at 90-h APF (Fig 5A, 5C, 5E, 5G, 5I and 5K). Analysis of myofibril and sarcomere morphology by staining for actin and Myosin heavy chain (Mhc) showed that all *sqh* and *zip* knock-down fibers contain prominent actin accumulations (Fig 5B, 5D, 5F, 5H, 5J and 5L), which are often a landmark of nemaline myopathies [27,28]. Additionally, sarcomere length is significantly reduced in *sqh* and *zip* knock-down myofibrils (Fig 5M). Together, these findings demonstrate that nonmuscle myosin indeed plays an essential role during myofibril formation or maturation.

**Fig 6:**
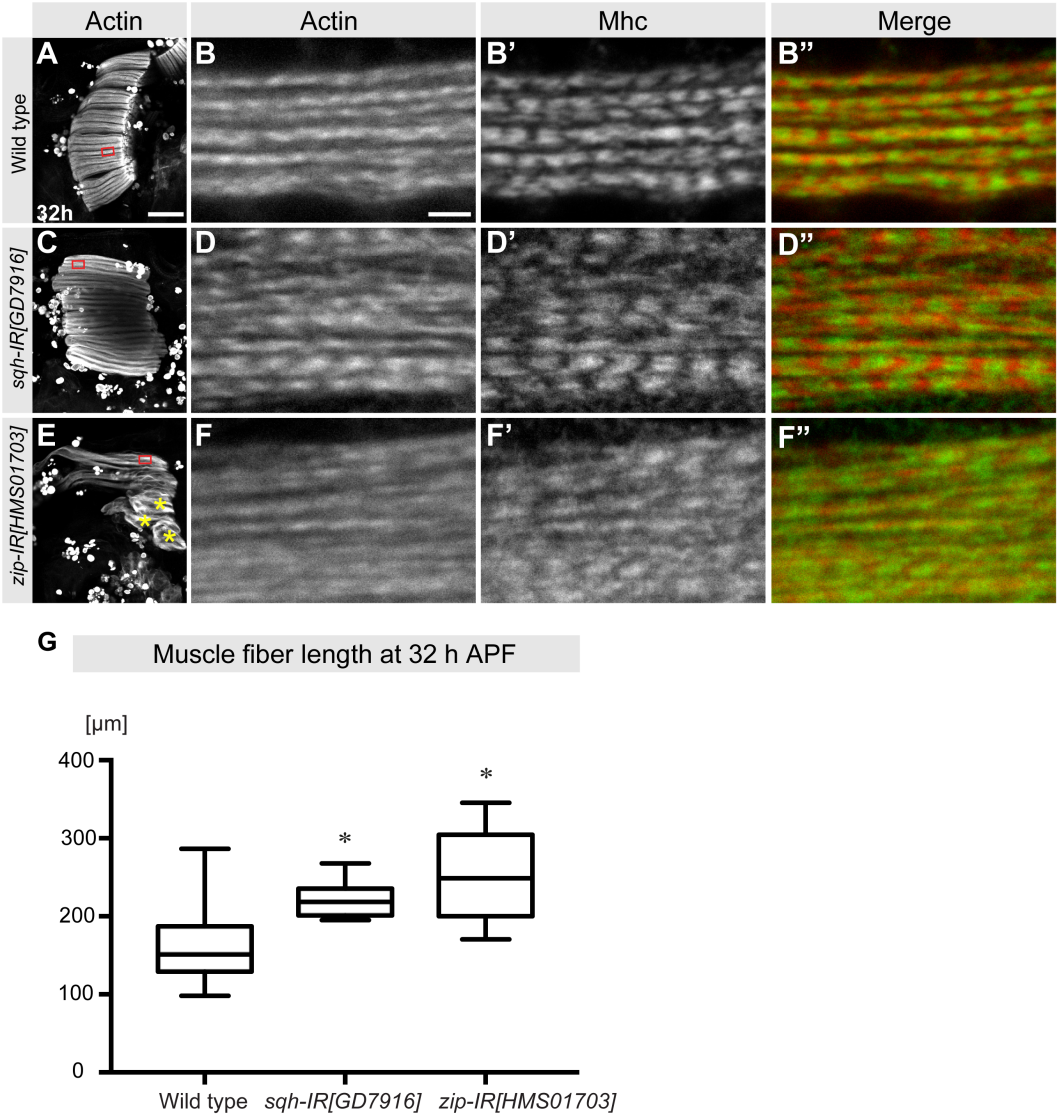
Nonmuscle myosin II is essential for myotube compaction. (A–F) Flight muscle fibers of wild-type 32-h APF pupae and of the indicated *sqh* and *zip* knock-down (hairpins expressed with *Mef2*-GAL4) were stained with phalloidin (red) and anti-Mhc (green) and observed by confocal microscopy (panels A, C, and E). Unattached rounded fibers after knock-down of *zip* are labeled with yellow asterisks (panel E). Red boxes indicate the positions of the high-magnification images that display the immature myofibrils. Scale bars represent 100 μm (panels A, C, and E) and 2 μm (panels B, D, and F). (G) Quantification of muscle fiber length at 32-h APF of the indicated genotypes. Plots represent the average sarcomere length per pupa shown within the whiskers (minimum to maximum), with 50% of the values shown within the boxplots (25% to 75%) and the median indicated as a line. *p*-values: *P < 0.05 (nonparametric Mann-Whitney U test; see S1 Data for primary data). APF, After Puparium Formation; Mhc, Myosin heavy chain; *sqh*, *spaghetti-squash*; *zip*, *zipper*.

We further investigated the stage of myofibril initiation at 32-h APF using the *sqh-IR[GD7916]* and *zip[HMS01703]* RNAi lines, which resulted in strong myofibril and sarcomere length phenotypes at 90-h APF. We found that *sqh* knock-down myofibers have attached to tendons at 32-h APF. However, they are overly long (Fig 6A, 6C and 6G). *zip* knock-down also results in some abnormally long fibers (Fig 6A, 6E and 6G) and in some unattached round fibers, which supposedly degenerate later, leading to the missing fiber phenotype at 90-h APF (Fig 5K). The myofibrils of the overly long fibers appear largely normal when stained for actin and Mhc (Fig 6B, 6D and 6F). These findings suggest that nonmuscle myosin contributes to the generation of mechanical tension before 32-h APF, which drives myofiber compaction and thus myofiber shortening during attachment maturation [11].

### Nonmuscle myosin II and mechanical tension are required for actin ordering during myofibril initiation

**Fig 7:**
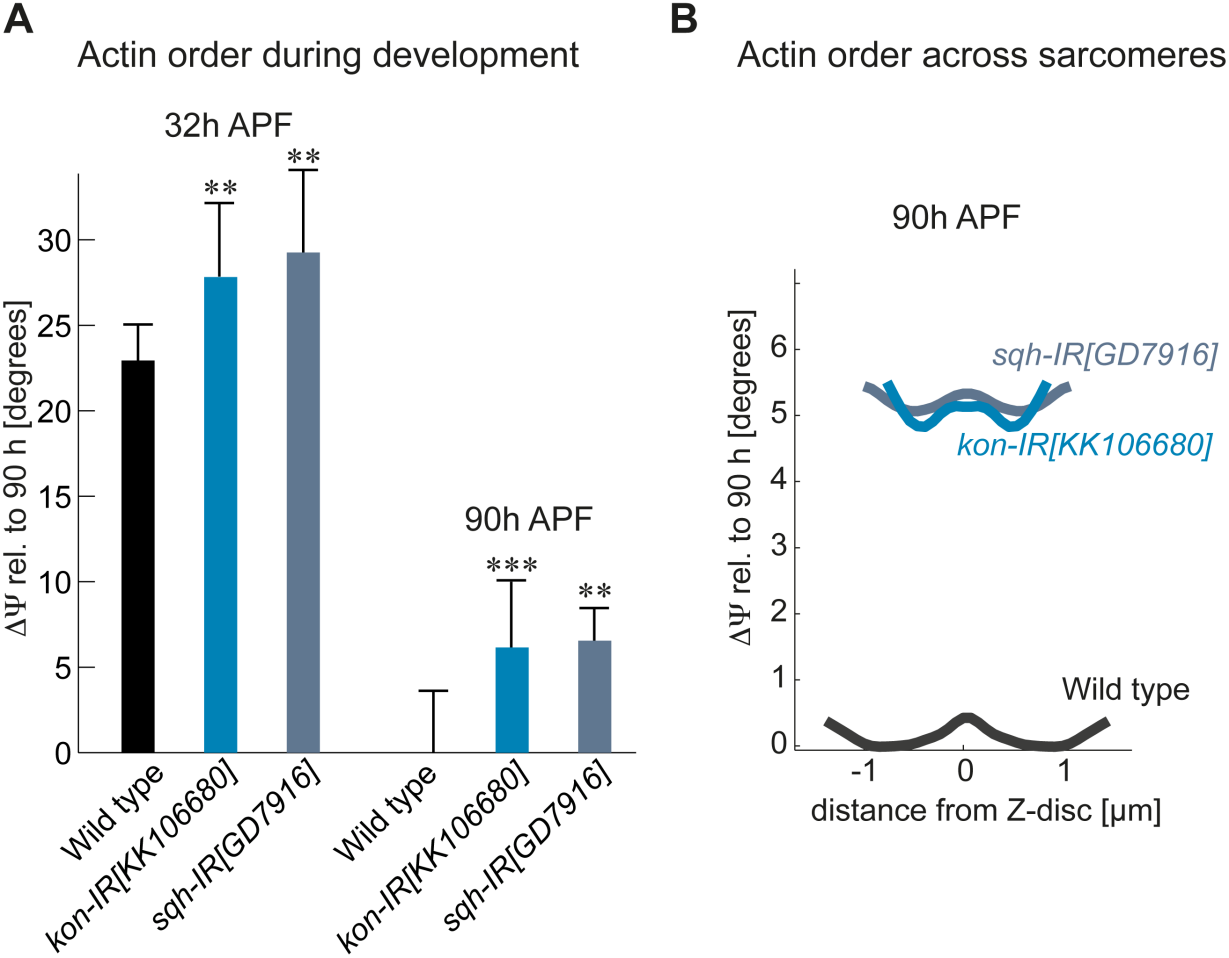
Nonmuscle myosin II and tension are required for molecular actin ordering during myofibril initiation. (A) Actin molecular disorder angles ΔΨ for *kon* and *sqh* knock-down muscles at 32-h and 90-h APF, relative to wild-type 90-h APF. Error bars represent SD. Note the higher actin disorder ΔΨ after *kon* or *sqh* knock-down at 32-h APF; however, notice the largely preserved actin order buildup at 90-h APF. *p*-values: **P < 0.01; ***P < 0.001 (nonparametric Mann-Whitney U test; see S1 Data). Error bars represent SD. (B) Molecular actin order angle profiles along the averaged sarcomere length for *kon* knock-down (light blue), *sqh* knock-down (slate blue), and wild-type control (black) at 90-h APF. Values are relative to the minimum of the wild-type profile. See S1 Data for primary data. APF, After Puparium Formation; *kon*, *kon-tiki*; *sqh*, *spaghetti-squash*.

In vitro and in vivo studies have shown that myosin contractility plays a central role for actin network organization [29,30]. We were therefore surprised to see a rather normal gross actin organization upon reduction of nonmuscle myosin II function in myofibrils at 32-h APF. To gain more detailed insight into the molecular organization of actin in these immature myofibrils, we applied our polarization-resolved microscopy technique. We found that molecular actin order is reduced by 6° after a knock-down of *sqh* in the immature myofibrils, demonstrating that nonmuscle myosin is indeed required for molecular order gain during early stages of myofibrillogenesis (Fig 7A).

As reducing nonmuscle myosin function resulted in a myofiber compaction defect and thus likely in a reduction of mechanical tension in the muscle, we investigated a different genetic alteration that results in a similar phenotype. Reducing the levels of the cell surface protein Kon-tiki (Kon) by RNAi leads to delayed myotube–tendon attachment, overly long muscle fibers, and therefore likely also a reduction of tension buildup. These fibers also show myofibrillogenesis defects [11]. We found a similar reduction in molecular actin order generation by 5° in the *kon* knock-down muscle as in the *sqh* knock-down muscle (Fig 7A). This strongly suggests that nonmuscle myosin and mechanical tension are both required for molecular actin ordering during myofibril initiation. By analyzing the mature 90-h APF muscles of the *sqh* and *kon* knock-down pupae, which both contain too short but regular sarcomeres (Fig 5 and [11]), we find that most of the order gain during sarcomere maturation occurs normally in these muscles (Fig 7A and 7B). Thus, despite reduced sarcomere length, actin does strongly gain molecular order in these shorter sarcomeres.

### Muscle myosin motor-independent gain of actin order during sarcomere maturation

As we found a strong gain of molecular actin order during sarcomere maturation under conditions of reduced nonmuscle myosin function, we suspected an important role for muscle myosin in actin ordering. *Drosophila* contains a single myosin heavy chain gene, *Mhc*. *Mhc* null mutations are embryonic lethal. However, the *Mhc[10]* allele carries a splice site mutation that specifically eliminates *Mhc* RNA and consequently Mhc protein isoforms from adult flight muscles [31–33]. Electron microscopic analysis had shown that the *Mhc[10]* flight muscles lack any detectable thick filaments [31,33]. Using mRNA sequencing (mRNA-Seq), we confirmed that the flight muscle–specific isoforms affected by the *Mhc[10]* splicing mutation are indeed the major *Mhc* isoforms expressed during myofibril formation and maturation stages (S2 Fig). These *Mhc[10]* mutants are homozygous viable and flightless and thus perfectly suited to quantify actin order during flight muscle development.

**Fig 8:**
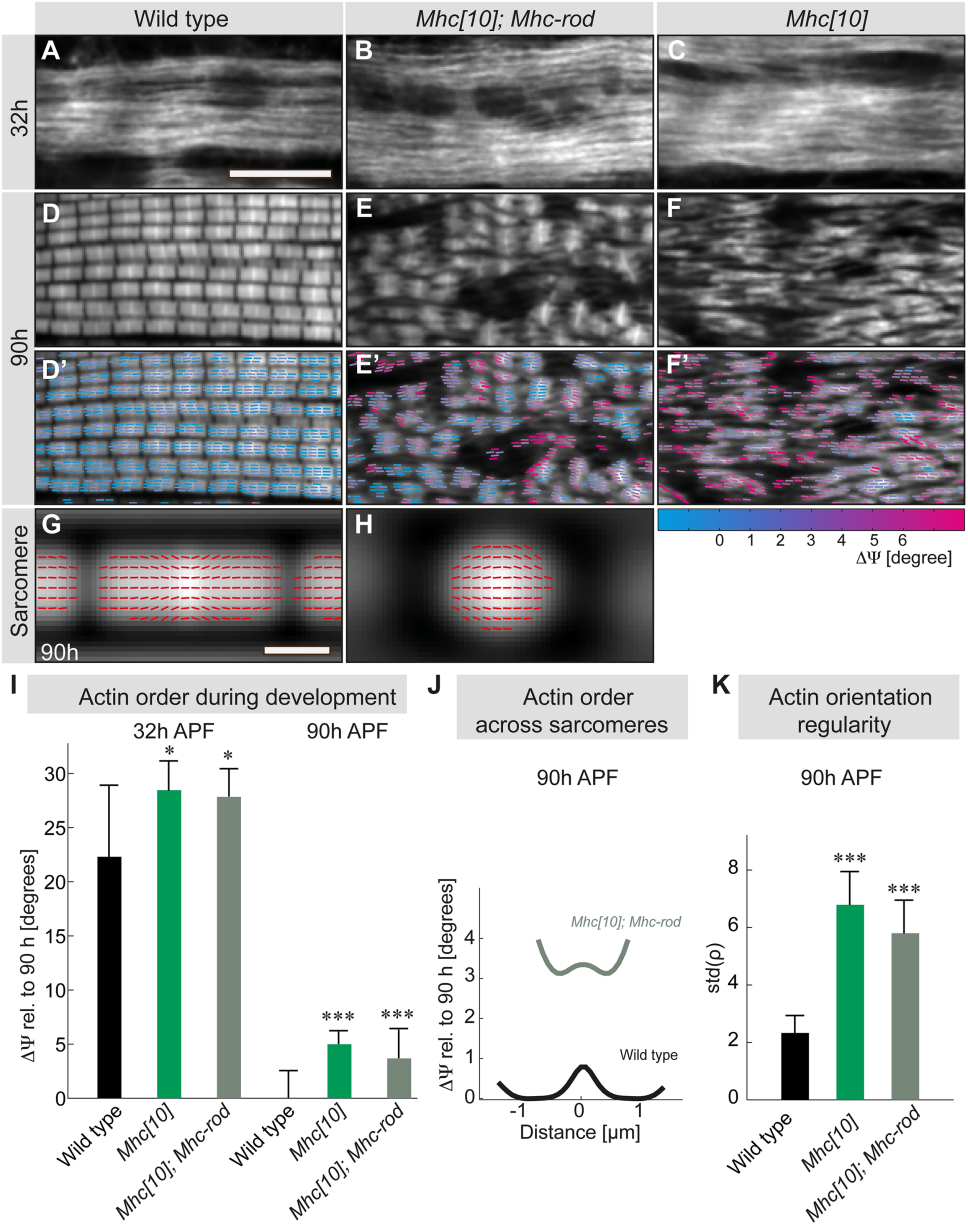
Muscle myosin is largely dispensable for order buildup during sarcomere maturation. (A–F) Flight muscle myofibrils at 32-h and 90-h APF of wild type (panels A, D), *Mhc[10];Mhc-rod* (panels B, E), and *Mhc[10]* (panels C, F) stained with Alexa488–phalloidin. (D’–F’) Sticks represent the local actin orientations (ρ) with a color scale of the local disorder angles compared with wild-type 90-h APF. Scale bar represents 10 μm. (G–H) Grey maps represent averaged actin intensity of wild-type (panel G) and *Mhc[10];Mhc-rod* sarcomeres (panel H). Red sticks show the local actin orientation (ρ). Stick angles have been amplified 50 times relative to horizontal axis. Scale bar represents 1 μm. (I) Actin molecular disorder angles ΔΨ relative to the control (90-h APF wild type) for wild-type, *Mhc[10]*, and *Mhc[10];Mhc-rod* at 32-h and 90-h APF. *p*-values: *P < 0.05; ***P < 0.001 (nonparametric Mann-Whitney U test; see S1 Data). Error bars represent SD. (J) Actin molecular disorder angle profile along the averaged sarcomere length for the wild-type control (black) and *Mhc[10];Mhc-rod* (grey-green) at 90-h APF. Values are relative to the minimum of the wild-type profile. (K) Variation of the local actin orientations ρ in the sarcomeres of wild-type, *Mhc[10]*, and *Mhc[10];Mhc-rod* pupae at 90-h APF. The variation is displayed as the averaged SD of ρ of each pixel compared with its neighbors (see Materials and methods for details). Note the increased variation in the *Mhc[10]* and *Mhc[10];Mhc-rod* muscles. See S1 Data for primary data. APF, After Puparium Formation; Mhc, Myosin heavy chain.

In addition to the *Mhc[10]* allele, we also analyzed muscles in which we re-expressed a headless *Mhc* rod construct using the flight muscle–specific *Actin88F* promoter in an *Mhc[10]* mutant background. This genetic set-up was shown to enable the assembly of actin filaments with myosin rods to sarcomere-like structures of variable length [32]. It results in a flight muscle compaction defect that is comparable to the nonmuscle myosin knock-down at 32-h APF [11], suggesting that Mhc also contributes to force generation during myofibril assembly. This notion was corroborated by the finding that molecular actin order in *Mhc[10]* and *Mhc[10];Mhc-rod*-expressing flight muscles at 32h APF is reduced to a similar extent as in the nonmuscle myosin or *kon* knock-down (Figs 7, 8A–8C and 8I). These results confirm the sensitivity of our order measurements that detect clear differences, although myofibril morphology—as assessed by regular spinning disc imaging—appears comparable.

At 90-h APF, we reproduced the known severe myofibril and sarcomere maturation defect in the *Mhc[10]* and *Mhc[10];Mhc-rod*-expressing flight muscles (Fig 8D–8F) [32,33]. Although the presence of Mhc rods supports the formation of bipolar actin filaments surrounding bright Z-disc–like structures, these filaments are poorly connected compared with the ordered wild-type myofibrils (Fig 8D and 8E). Without the Mhc rod, the actin pattern appears drastically disorganized with no detectable periodicity (Fig 8F). These results confirm that muscle myosin motor activity is required for proper myofibril maturation.

We were surprised to find that actin order buildup is still largely supported in these *Mhc[10]* and *Mhc[10];Mhc-rod*-expressing flight muscles during stages of myofibril and sarcomere maturation. At 90-h APF, the molecular disorder ΔΨ is only increased by 5° in *Mhc[10]* and by 4° in *Mhc[10];Mhc-rod*-expressing flight muscles compared with wild-type controls (Fig 8I). We have been able to automatically segment sarcomere-like structures in the *Mhc[10];Mhc-rod*-expressing flight muscles and find that the relative molecular disorder at the Z-disc compared with wild type is less than 3°. However, at the edges of the sarcomeres, order decays more rapidly than in wild type (Fig 8J). We conclude from these data that high molecular actin order in developing muscles can be achieved without the motor activity of muscle myosin.

The overall organization of myofibrils is drastically affected in *Mhc[10]* and in *Mhc[10];Mhc-rod* flight muscles compared with controls (Fig 8D–8F). Accordingly, we find that the collective orientation of the actin molecules per pixel as measured by ρ shows larger variation in *Mhc[10]* and in *Mhc[10];Mhc-rod* flight muscles at the fiber scale (Fig 8D’–8F’). We quantified this variation of ρ by calculating its SD and comparing each pixel with its neighbors in a 10 μm × 10 μm square. This demonstrates that the overall actin filament orientation is more irregular in *Mhc[10];Mhc-rod* flight muscles compared with controls (Fig 8K), suggesting that the myofibril self-organization or myofibril maturation does require muscle myosin to be coordinated across the entire muscle fiber. It also validates that our approach can detect both local molecular actin order in each pixel and global regularity of the actin orientation in the entire system, here the myofibrils. In summary, polarization-resolved microscopy enabled us to demonstrate that Mhc—and in particular the motor activity of Mhc—is largely dispensable for local order generation of actin during sarcomere maturation. However, it is required for global coordination of regular myofibril maturation throughout the developing muscles.

## Discussion

Mature muscles possess a pseudocrystalline order of their actomyosin filaments, which needs to be built up during muscle development. Although myofibrillogenesis is generally important and has been studied for many years, its molecular mechanism is not well understood. One limitation of past studies was the difficulty of quantifying molecular order of sarcomeric protein components in vivo. Here, we have applied polarization-resolved microscopy to quantify actin order buildup during myofibrillogenesis using the *Drosophila* indirect flight muscle as an in vivo model system. This model is particularly well suited for studying molecular order buildup because insect flight muscle is considered the most ordered muscle in nature—it is even a suitable sample for X-ray structural studies [22,23].

Using polarization-resolved microscopy, we found that molecular actin order starts to build up between 22-h and 32-h APF after myotubes have attached to tendons, coinciding with the assembly of immature myofibrils. We discovered that actin molecular order is built up simultaneously throughout the entire large muscle cell. This finding challenges in vitro data, which showed different myofibril organization levels in the same cell at the same time and concluded a stepwise myofibril assembly as proposed in the premyofibril model [14–16,25]. Our finding does not refute the premyofibril model; however, it does strongly support a self-organization mechanism of myofibril assembly, coordinating the assembly throughout the entire muscle cell. The same myofibril self-organization was recently also found in cultured cardiomyocytes, when cardiomyocytes were allowed to generate forces by the formation of cell-matrix adhesions [34]. In mutants with reduced muscle attachment and thus reduced force generation, order buildup is delayed, corroborating the hypothesis that mechanical tension is important for coordinating the self-organization process [3,11,34].

Mechanistically, we demonstrated that nonmuscle myosin and muscle myosin play important roles for actin ordering during the formation of immature myofibrils at 32-h APF. This is an important finding because it adds functional genetic evidence to the previously suggested early role of nonmuscle myosin during “premyofibril” formation in vertebrate myogenesis, which was largely concluded from myosin drug experiments performed with in vitro culture models [14,35]. It also provides insight into the force generation mechanism during myofibril formation, which requires stable attachment to tendons, nonmuscle myosin, and muscle myosin activity because interfering with either one results in overly long muscle fibers, with defects in actin order buildup at 32-h APF.

We found that most actin order buildup is completed by 48-h APF, at which stage 2.1-μm–long, regular, immature sarcomeres are present. This is a remarkable finding considering that, during the following increase in sarcomere length and in particular sarcomere diameter, the actin order gain is rather small. This is well supported by our electron microscopy images showing the regular actomyosin arrays of immature sarcomeres at 48-h APF. Mechanistically, this finding suggests that newly added actin filaments, which mediate the longitudinal growth of the sarcomere, are added in a way that respects the already present high molecular order. It also suggests that the newly added actin filaments at the periphery of the myofibrils that mediate lateral growth to expand the myofibril diameter [36] are either already ordered or gain molecular order quickly. These findings are consistent with a model of myofibrillogenesis that proposed that muscle myosin containing thick filaments can assemble directly with cross-linked thin filaments composed of α-actinin, actin, and titin (I-Z-I bodies) [37,38]. This assembly is likely to occur laterally on the growing preassembled immature myofibril using the existing ordered actin and myosin filaments as a template. Concomitantly, both filament types grow in length to reach 3.1-μm sarcomere length at 90-h APF. If the actin order during assembly of immature myofibrils at 32-h APF is compromised, either by reducing nonmuscle myosin or mechanical tension, the defect cannot be rescued during the following sarcomere maturation steps, resulting in short nonfunctional sarcomeres at 90-h APF [11].

We found that the buildup of molecular actin order in flight muscles after 32-h APF is largely independent of muscle myosin function. In both the *Mhc[10]* splice mutant muscles as well as in *Mhc[10];Mhc-rod*-expressing muscles, actin is ordered almost as well as in the wild-type muscle, at least per pixel, which is the detection volume of our microscopy method. We can rule out the trivial explanation that other muscle myosin isoforms are expressed in the *Mhc[10]* mutant flight muscle because the point mutation affects the isoforms that are predominant in the adult flight muscles as well as during flight muscle development; in addition, western blotting showed that more than 95% of Mhc protein is lost in *Mhc[10]* in adult flight muscles [33]. Electron microscopy analysis showed that, in adult *Mhc[10]* flight muscles, Z-disc–like structures can be found—which, in some cases, are connected to periodic structures that nevertheless entirely lack thick filaments [33]. A second argument against remaining Mhc protein activity in the *Mhc[10]* mutants is that the *Mhc[10]* phenotype is not enhanced by the expression of the headless myosin rod. If this construct is expressed in wild-type muscle, it creates a dominant negative phenotype, resulting in the fragmentation of the myofibrils [32]. In contrast, expression of the Mhc rod in the *Mhc[10]* mutant results in a milder myofibril phenotype with clearly visible, sarcomere-like structures that are often connected with the headless myosin rods [32]. Together, these data provide strong arguments that muscle myosin is largely dispensable for local order generation of actin during myofibril and sarcomere maturation. Thus, other actin cross-linkers—like α-actinin, the ZASP family of proteins, and in particular the different *Drosophila* titin isoforms Kettin, Sallimus, or Projectin—are likely candidates contributing to actin ordering from the Z-disc. This would fit our observation that actin order is higher at the Z-disc in forming sarcomeres at 48-h APF. All these Z-disc proteins play an important role during sarcomerogenesis [26,39–41], but testing their role in molecular actin or myosin order buildup will be a challenge for the future.

Despite the buildup of local molecular actin order in the muscle myosin mutants, we found that the actin order orientation along the muscle and sarcomere axis is severely perturbed. This demonstrates that myosin motor activity is required to globally order the actin filaments along the future contraction axis of the muscle fiber. This is in accordance with recent data demonstrating that myosin-mediated muscle contractions are required for higher muscle-fiber–wide orientation of the actomyosin filaments resulting in cross-striated muscle fibers, with all myofibrils aligned along the lateral muscle axis [42]. These myosin-dependent higher order assemblies are not restricted to muscle cells but can also be found in stress fibers of nonmuscle cells [43], again suggesting that myosin contractility is required for the global self-organization of a regular periodic actomyosin pattern throughout the cell [6].

Taking these data together, we suggest the following updated model for in vivo myofibrillogenesis and sarcomere maturation.

1. Immature myofibrils assemble simultaneously throughout the entire muscle fiber. This step requires muscle attachment and the buildup of mechanical tension, which coordinates the self-organization of actin, myosin, and titin filaments into early immature myofibrils. This first step generates significant molecular order of actin, which depends on the function of both nonmuscle and muscle myosin, both of which contribute to the mechanical force generation resulting in myofiber compaction.
2. During sarcomere maturation, sarcomere length and diameter increase simultaneously across all sarcomeres of the muscle cell, again coordinated by a self-organization mechanism. The incorporation of new filament subunits maintains the preformed high molecular order of actin and myosin filaments.
3. Muscle myosin motor activity is required for the global self-organization of the periodic sarcomeric series into linear periodic myofibrils but is largely dispensable for local molecular actin ordering, which must therefore be achieved by alternative mechanisms. As most sarcomeric components as well as the global organizational principles of sarcomeres and myofibrils are conserved from flies to mammals, this model should also be relevant to mammalian muscle morphogenesis.

## Materials and methods

### *Drosophila* strains

All *Drosophila* work was performed at 27 °C to enhance GAL4 activity. Muscle-specific expression was achieved using *Mef2*-GAL4 [44]. *sqh* (KK109493, GD7916), *zip* (GD7819, HMS01618, HMS01703), and *kon* (KK106680) RNAi lines were obtained from VDRC [45] and Bloomington stock centers [46]. *Mhc[10]* mutants were obtained from S. Bernstein [33]. Flight muscle–specific headless myosin flies (*Mhc[10]; Mhc rod*) were generated by expressing headless Mhc (V138 isoform) under *Actin88F* control in an *Mhc[10]* mutant [32].

### Fixation and staining of flight muscles

Pupae were staged and dissected as described [47]. Briefly, pupae were fixed for 15 min in 4% paraformaldehyde (PFA) in PBST (PBS plus 0.3% TritonX) at room temperature (RT) and washed in PBST. Pupae 90-h APF were then cut sagittally using a microtome blade. Dissected pupae were blocked for 30 min with normal goat serum (1:30), stained with mouse anti-Mhc 1:100 (J. Saide, Boston University, Boston, MA) overnight (ON) at 4 °C, and washed 3 times in PBST. Secondary antibodies (1:500) and rhodamine phalloidin (1:500) (both from Molecular Probes, Eugene, OR) were added for 2 h at RT, followed by 3 washing steps in PBST before samples were embedded in Vectashield. For the polarization-resolved microscopy, pupae were only stained with Alexa488–phalloidin (Molecular Probes) for 2 h at RT, washed, and embedded. Images were acquired with a Zeiss LSM 780 and processed with Fiji [48] and Photoshop.

### Sarcomere length quantification

Using Fiji [48], a straight line was drawn over a myofibril following the phalloidin labeling, and an Mhc plot profile was generated. From the plot, the distances between the intensity peaks were calculated. All sarcomere length values per pupa were averaged, and thus one value per pupa was used for the statistics. Nine to 10 pupae were quantified for each genotype (S1 Data).

### Electron microscopy

The flies were staged and dissected as described in [47], with the modification that 0.1 M phosphate buffer (PB), pH 7.0, was used as the dissection solution. After dissection, samples were fixed at RT in 4% PFA and 0.5% glutaraldehyde in 0.1 M PB for 3 h. The samples were transferred to 4 °C ON. On the next day, samples were fixed in 2% OsO_4_ in 0.1 M PB for 1 h on ice in the dark. Samples were washed 5 times with ddH_2_O and stained en bloc with 2% uranyl acetate (UA) at RT for 30 min in the dark. After another washing step (5 times ddH_2_O), the samples were dehydrated in a graded ethanol series (30%, 70%, 80%, 90%, 95%) each on ice for 3 min and 2 times 100% ethanol at RT for 5 min. Following dehydration, samples were incubated in pure propylene oxide (PO) 2 times for 15 min and then transferred to an epon PO mixture with a ratio of 1:1 ON to allow resin penetration. After the PO was removed by slow evaporation over 24 h, samples were embedded into freshly prepared epon (polymerization at 60 °C for 24 h). Sections of 90 nm were cut on a Leica UC7 microtome and were additionally stained with 2% UA for 30 min and 0.4% lead citrate for 3 min to enhance the contrast. Images were acquired with a Zeiss EM 900 (80 kV) using a side-mounted camera from Osis.

### RNA-Seq

mRNA-Seq was performed on dissected flight or leg muscles at 24-h, 30-h, 48-h, 72-h, and 90-h APF pupae or 1-d adults. Samples were prepared as previously described [24] and sequenced on an Illumina Hi-Seq2000 (VBCF, Vienna, Austria). Reads were mapped to the *Drosophila* genome (BDGP6.80 from ENSEMBL) using STAR, sorted and indexed using samtools, normalized by library size and read-counts, converted to bigwig files, and visualized on the UCSC server. Detailed analysis of the mRNA-Seq time course has been described [49].

### Polarization-resolved fluorescence microscopy

The optical set-up, described in detail in [50], is based on a confocal spinning disk unit (CSU-X1-M1; Yokogawa, Tokyo, Japan) connected to the side port of an inverted microscope (Eclipse Ti-U; Nikon, Tokyo, Japan). The laser excitation is provided by a polarized 488-nm continuous laser (Sapphire 488–20; Coherent, Santa Clara, CA) whose power is controlled. The laser beam is sent into an electro-optic modulator (EOM) (Pockels cell, No 28-NP; Quantum Technology, Lake Mary, FL) followed by a quarter wave plate (WPQ05M-488; Thorlabs, Newton, NJ) for production of a linear rotating polarization. A polarization distortion compensator is used to compensate for ellipticity and diattenuation produced by the optics in the excitation path towards the microscope objective, which are characterized preliminarily [50]. The beam is then expanded using a 10× telescope (BE10; Thorlabs) and sent directly to the microlens array of the CSU by reflection in its dichroic mirror (Di01-T488-13×15×0.5; Semrock, Rochester, NY). An objective lens (Nikon Plan Apo VC 60×, N.A. = 1.2, water immersion) is used for excitation and light collection. The detection is then filtered (bandpass 525/45) and imaged onto a camera (iXon 897 EMCCD, 512×512 pixels; Andor, Belfast, United Kingdom). The microlens and pinhole array of the CSU disks rotate synchronously at a speed of 1,800 rpm, while the EMCCD and EOM are synchronized to ensure a fast stack recording for a given number of incident polarization [50]. A frame rate of 30 ms per image and 10 polarization angles measured leads to a typical rate of 1 polarization stack recorded per second.

### Molecular order angle

Inside the confocal volume, each Alexa488–phalloidin molecule exhibits an absorption dipole vector ***μ***_*abs*_ with an orientation (*θ*, *φ*) in 3D. Fluorescence is generated from the coupling of these dipoles with the incident linear polarization ***E***(*α*), which makes an angle *α* with the horizontal axis *x* of the sample plane. The recorded fluorescence intensity is proportional to the absorption probability *P*_*abs*_ = |***μ***_*abs*_(*θ*, *φ*).***E***(*α*)|^2^. The total intensity buildup from the incoherent emission from all molecules, during the time of the measurement over which they might fluctuate in orientation, results in an angular integration over all angles explored in time and space: *I*(*α*) = *∫∫*|***μ***_*abs*_(*θ*, *φ*).***E***(*α*)|^2^ sin *θdθdφ*. This expression shows that the signal is modulated in *α* when the absorption dipoles of the fluorescence probes are aligned, e.g., when they do not experience an isotropic distribution. We assume that the orientations explored by molecular dipoles are constrained within an angular cone total aperture angle *ψ*, oriented in the sample plane along the direction *ρ* relative to X (Fig 1A). Physically, *ψ* determines the degree of angular variations present within the focal spot at a given pixel position. This angle, denoted “molecular order”, encompasses the orientation variations among probes (related to the static organization of actin filaments) as well as their time angular fluctuations (related to the degrees of freedom relative to their binding site). *ρ*, on the other hand, determines the preferential orientation of the probes. *ρ* and *ψ* thus permit us to quantify the full information on molecular organization at each pixel of an image (typically of size 300 nm × 300 nm).

In practice, (*ρ*, *ψ*) are deduced from the measurement of the intensity modulation *I*(*α*). This is done by decomposing the dependence of the intensity *I*(*α*) as a function of (*ρ*, *ψ*) in a modulation form: *I*(*α*) = *a*_0_ + *a*_2_(*ρ*, *ψ*) cos 2*α* + *b*_2_(*ρ*, *ψ*) sin 2*α*. *a*_2_(*ρ*, *ψ*) and *b*_2_(*ρ*, *ψ*) are the intensity modulation coefficients. Their relation to (*ρ*, *ψ*) are computed numerically, accounting for possible polarization distortions of ***E***(*α*), which permit us to retrieve (*ρ*, *ψ*) from the measurement of (*a*_2_, *b*_2_) at each pixel position [18]. In practice, these coefficients are measured from the computation of *a*_2_ = 2/*a*_0_ ∑_*k*_ *I*(*α*_*k*_) cos 2*α*_*k*_ and *b*_2_ = 2/*a*_0_ ∑_*k*_ *I*(*α*_*k*_) sin 2*α*_*k*_, using *a*_0_ = ∑_*k*_ *I*(*α*_*k*_) the total intensity and *a*_*k*_ the angles used for the polarization-resolved measurements (typically *k* = 1..10 and *α*_*k*_ = 0°…180°). (*ρ*, *ψ*) are finally represented in a map that combines molecular order and orientation.

### Threshold image

After having manually selected the area of interest in a raw intensity image, we applied a smooth filter on the raw image and divided the raw image by the filtered one. Local actin-rich areas compared to their vicinity result in a value greater than 1. A threshold around 1 permits us to obtain a binary mask corresponding to actin filaments. At 48-h, 72-h, and 90-h APF, the threshold discards M-lines due to low actin signal from the binary mask.

### Automatized segmentation method

We detected all objects (connected areas) within the binary mask. We then used the method “regionprops” of Matlab to fit every object as an ellipse and detected sarcomeres by applying criteria on the geometrical property of the object. These criteria are based on the convexity of the object, the area of the object, and the ratio between the major and minor axes of the fitting ellipse. Minimal and maximal values of each criterion depend on the age of the pupae. This process has been applied to get data for Figs 2G–2J, 4C–4F, 7B, 8G, 8H, 8J and 8K.

### Averaged actin molecular order angle

Applying a binary mask on its corresponding intensity image, we obtained a mean image value. An individual hemithorax value is given by an average of several mean image values. Number of recorded images by hemithorax is between 10 and 20 (except for 90-h APF *kon-IR [KK106680]*, which were 5 images). We finally calculated the averaged actin molecular disorder angle of the population (between 5 and 31 hemithoraxes per population). This process has been applied to get data from Figs 2K, 7A and 8I.

### Averaged sarcomere map and profile

To reconstitute sarcomere maps, only the 20 most central sarcomeres were selected per image. This avoids optical aberrations that are more frequent at the border of the images. Averaged sarcomere profile corresponds to a 1-pixel–thick line along the center of the averaged sarcomere map. This process was applied to get data from Figs 2G–2J, 4C–4F, 7B, 8G, 8H and 8J.

### Rho variability

Discarding dark pixels within the threshold images, SD of the orientation angle *ρ* of each pixel relative to its 65 × 65 neighbors (10 μm × 10 μm) was measured. An average value was obtained for each image and then for each pupa. This process was applied to obtain data for Fig 8K.

### Primary data

All measured and calculated values as well as the sample numbers, statistics, and *p*-values are listed in S1 Data.

## Supporting information

S1 Fig Simultaneous order buildup during myofibril initiation. (A–E) Flight muscles of different pupae at 32-h APF stained with rhodamine–Alexa488. Overlaid longitudinal red lines (from one fiber end to the other) indicate positions at which molecular actin order was determined. The molecular order measurements are displayed in Fig 4B. Scale bars represent 20 μm. See S1 Data for primary data. APF, After Puparium Formation.
(TIF)

S2 Fig Developmental profile of *Mhc* splicing in flight muscle. Gene browse image with annotated Mhc isoforms containing exon 15a (green) or exon 15b (red) is shown. The position of the *Mhc[10]* point mutation in the splice acceptor site is indicated. Below are mRNA-Seq traces (RPKM values) from flight muscles of 24-h to 90-h APF (green) and adult leg muscles (orange). Note that exon 15a (green) is continuously expressed in flight muscles, whereas exon 15b is not detected, except for a minor peak at 48-h APF (red). Leg muscles express both exons. Below the sequencing traces are splicing junctions shown. APF, After Puparium Formation; Mhc, Myosin heavy chain; mRNA-Seq, mRNA sequencing; RPKM, Reads Per Kilobase of transcript per Million mapped reads.
(TIF)

S1 Data Primary experimental data. Table listing the individual experimental data for indicated figures in the different sheets. It includes the samples sizes, statistics, and *p*-values.
(XLSX)

S1 Table Genetic requirement of nonmuscle myosin II in muscle. Table lists the observed lethality and flightless phenotypes of various RNAi hairpins targeting *sqh* or *zip*, when expressed during muscle development with *Mef2*-GAL4. RNAi, RNA interference; *sqh*, *spaghetti-squash*; *zip*, *zipper*.
(TIF)

## Abbreviations

APF: After Puparium Formation
EOM: electro-optic modulator
F-actin: filamentous actin
Kon: Kon-tiki
Mhc: Myosin heavy chain
mRNA-Seq: mRNA sequencing
ON: overnight
PB: phosphate buffer
PFA: paraformaldehyde
PO: propylene oxide
RNAi: RNA interference
RT: room temperature
*sqh*: *spaghetti-squash*
UA: uranyl acetate
*zip*: *zipper*
