# Supplementary figures and images for: Polarization-resolved microscopy reveals a muscle myosin motor-independent mechanism of molecular actin ordering during sarcomere maturation
Source: PLoS Biol. 2018 Apr 27;16(4):e2004718. doi: 10.1371/journal.pbio.2004718 (PMC5955565; doi:10.1371/journal.pbio.2004718)

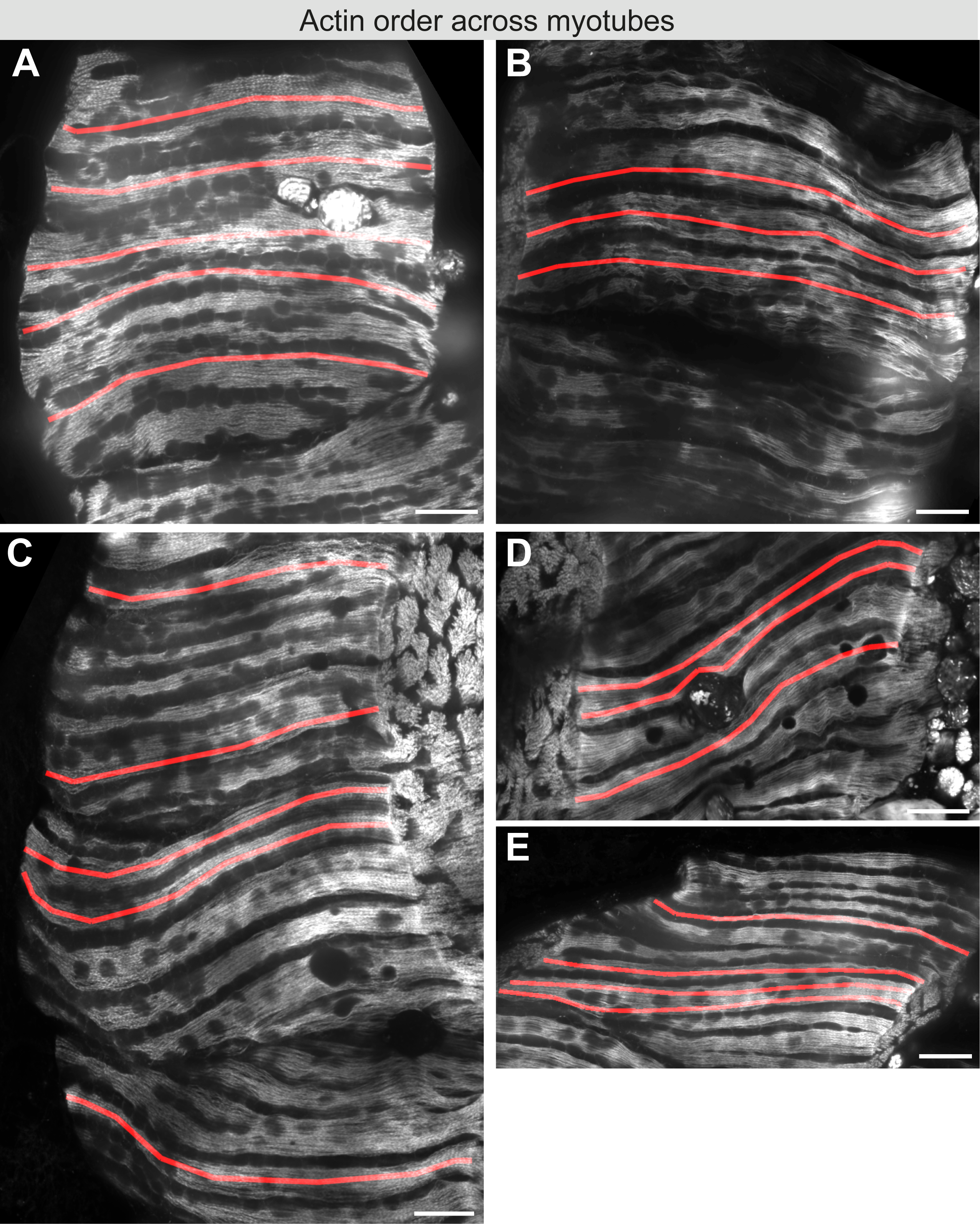

Supplement: S1 Fig — (A–E) Flight muscles of different pupae at 32-h APF stained with rhodamine–Alexa488. Overlaid longitudinal red lines (from one fiber end to the other) indicate positions at which molecular actin order was determined. The molecular order measurements are displayed in Fig 4B. Scale bars represent 20 μm. See S1 Data for primary data. APF, After Puparium Formation. (TIF) [file pbio.2004718.s001.tif]

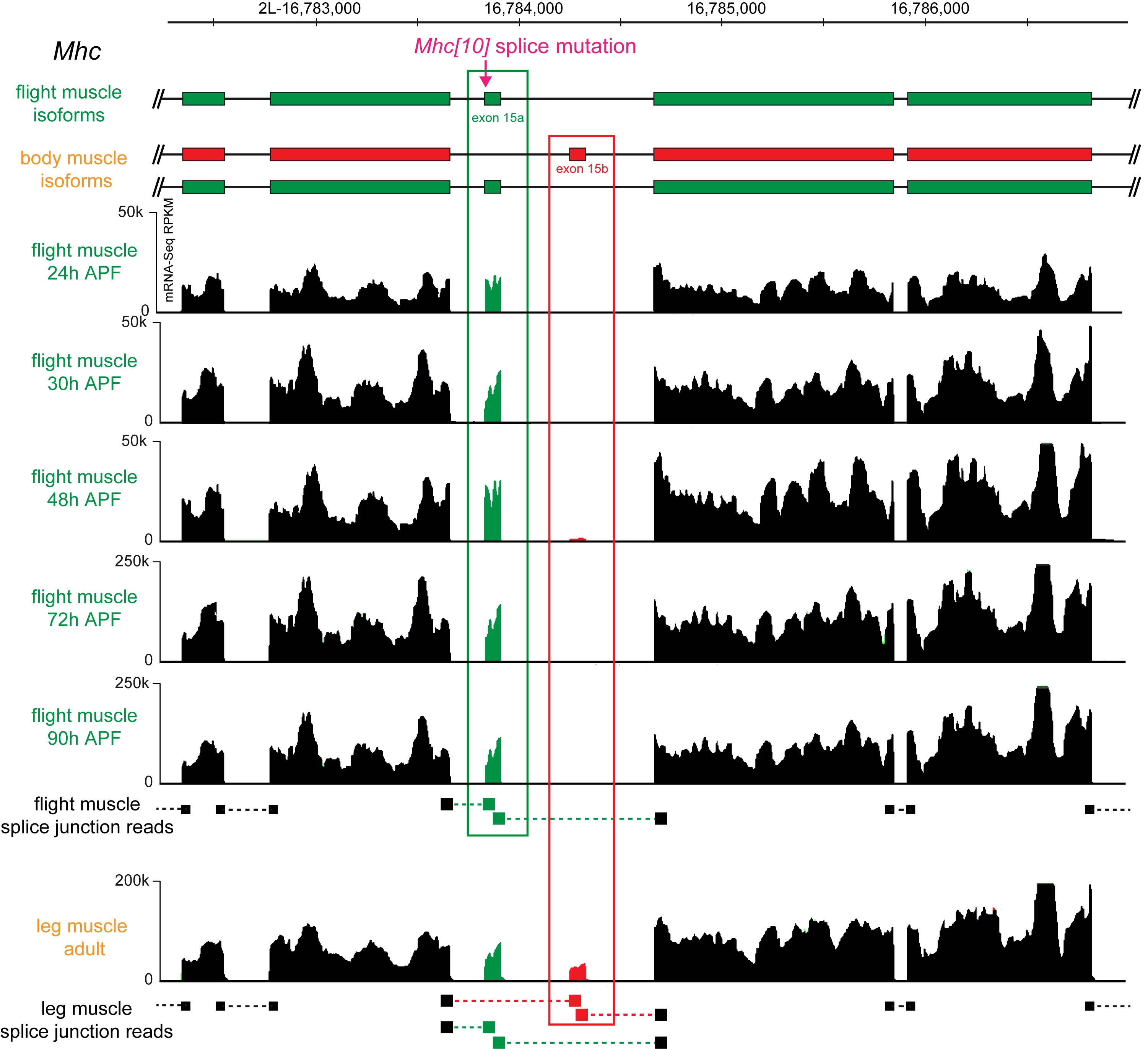

Supplement: S2 Fig — Gene browse image with annotated Mhc isoforms containing exon 15a (green) or exon 15b (red) is shown. The position of the Mhc[10] point mutation in the splice acceptor site is indicated. Below are mRNA-Seq traces (RPKM values) from flight muscles of 24-h to 90-h APF (green) and adult leg muscles (orange). Note that exon 15a (green) is continuously expressed in flight muscles, whereas exon 15b is not detected, except for a minor peak at 48-h APF (red). Leg muscles express both exons. Below the sequencing traces are splicing junctions shown. APF, After Puparium Formation; Mhc, Myosin heavy chain; mRNA-Seq, mRNA sequencing; RPKM, Reads Per Kilobase of transcript per Million mapped reads. (TIF) [file pbio.2004718.s002.tif]

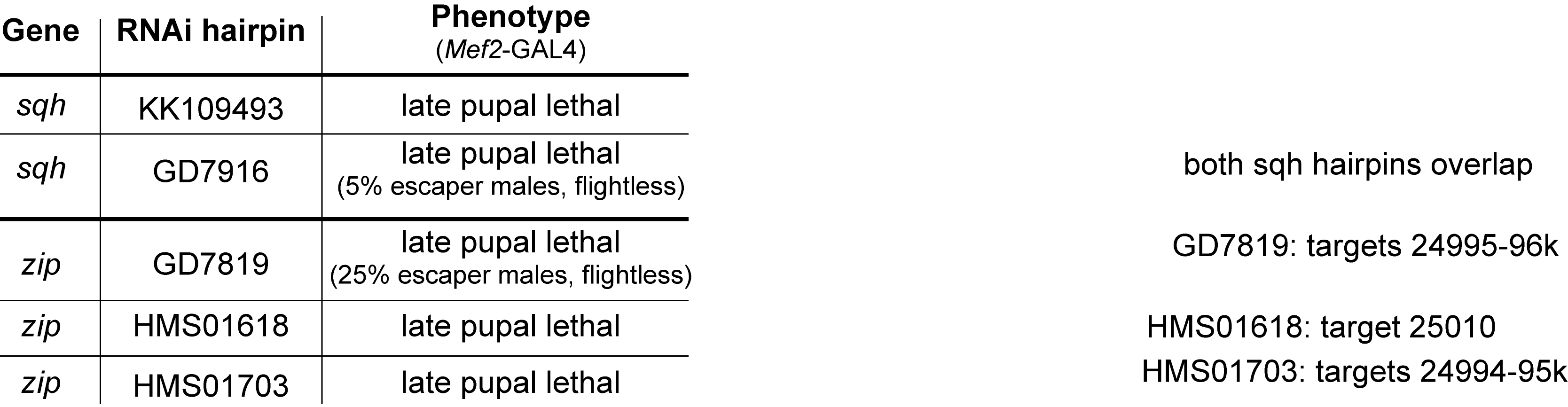

Supplement: S1 Table — Table lists the observed lethality and flightless phenotypes of various RNAi hairpins targeting sqh or zip, when expressed during muscle development with Mef2-GAL4. RNAi, RNA interference; sqh, spaghetti-squash; zip, zipper. (TIF) [file pbio.2004718.s004.tif]
